# Collision of Malignant Melanoma and Small Lymphocytic Lymphoma/Chronic Lymphocytic Leukemia: A Case Report and Literature Review

**DOI:** 10.3390/dermatopathology13040049

**Published:** 2026-10-15

**Authors:** Jing Zhou, Leila Cruthirds, Emily Cornelius, John Harrison Howard, Kurt Knowles

**Affiliations:** 1 Pathology Department, University of South Alabama Health, Moore Building, Room 138, Mobile, AL 36617, USA; 2 Surgical Oncology Department, University of South Alabama Health, Mobile, AL 36617, USA

**Keywords:** malignant melanoma, small lymphocytic lymphoma/chronic lymphocytic leukemia, collision tumor, pathogenesis, diagnostic pitfalls

## Abstract

Collision tumors are exceptionally rare. We report here a case of a cutaneous collision tumor composed of metastatic melanoma and small lymphocytic lymphoma (SLL). A 56-year-old man with a prior history of melanoma and chronic lymphocytic leukemia (CLL) presented with a new cutaneous nodule near the previous melanoma excision site on the right lower flank. Imaging identified a separate skin lesion in the right medial thigh. Histopathologic examination of both lesions revealed an infiltrative malignant melanoma in the flank and a melanoma with similar morphology in the medial thigh, adjacent to a dense, monotonous lymphoid infiltrate involving the subcutis and skeletal muscle. Immunohistochemical studies demonstrated that the melanoma cells were positive for SOX10, MART-1 and cyclin D1; the lymphoid population was positive for CD5, CD20, PAX5 and CD23, and negative for CD3, CD10 and cyclin D1. Given the known history, the combined morphologic and immunophenotypic findings support the diagnosis of a cutaneous collision tumor composed of melanoma and SLL/CLL. A review of the literature highlights the rarity of such cases and sheds light on current theories of pathogenesis, diagnostic pitfalls, and the mainstay treatment of this rare collision tumor.

## Simple Summary

Collision tumors occur when two different tumors are found together in the same tissue and are very rare. We report a 56-year-old man with a history of melanoma and chronic lymphocytic leukemia who developed a skin lesion containing both metastatic melanoma and small lymphocytic lymphoma/chronic lymphocytic leukemia. Examination of the biopsy showed two distinct tumor populations, which were confirmed by specialized staining. Our review of previously reported cases shows that this combination is uncommon, particularly in the skin. This case highlights the importance of carefully examining new skin lesions in patients with a history of melanoma, as the presence of a second cancer may otherwise be overlooked. Correctly identifying both tumors may affect molecular testing, staging, treatment decisions, and follow-up.

## 1. Introduction

Melanoma is the fifth most common cancer in the United States [1]. Small lymphocytic lymphoma (SLL) and chronic lymphocytic leukemia (CLL) are different manifestations of the same disease, with SLL in the nodal or solid phase and CLL in the leukemic phase. According to the most recent update of the SEER database, CLL is the most common leukemia [2]. A bidirectional association between malignant melanoma and SLL/CLL has been well established. According to one study, the incidence of lymphoma in melanoma patients is 0.3%, with SLL/CLL being the most common [3]. On the other hand, patients with SLL/CLL are at higher risk for second malignancies. Multiple population-based studies have shown a 2- to 8-fold greater risk of malignant melanoma development in patients with SLL/CLL [4,5,6]. However, the incidence of malignant melanoma and SLL/CLL collision tumors is extremely low.

Collision tumors are two morphologically and biologically distinct neoplasms coexisting at a common anatomic site. Since the first case report of melanoma and SLL/CLL collision tumors in an axillary lymph node by El Demellawy et al. [7] in 2007, only a limited number of additional cases have been reported, in addition to an image report at *BloodWork* [8]. Therefore, understanding of this phenomenon remains poor.

Here, we report a case of melanoma and SLL/CLL collision in the skin. We tabulate the literature, summarize the current theories of pathogenesis, and discuss diagnostic pitfalls and treatment strategies.

## 2. Materials and Methods

The clinical history of the patient was retrieved from the electronic medical record. Tissue samples were formalin-fixed and paraffin-embedded and stained with hematoxylin and eosin for histopathologic evaluation. Immunohistochemical staining for SOX10, MART-1, PAX5, CD3, CD5, CD10, cyclin D1, CD20, CD23, and Ki-67 was also performed to characterize the neoplastic cell populations. In addition, SOX10/CD5 dual staining was performed to highlight the sharp demarcation between the two components of the collision tumor.

A literature search was performed in PubMed through September 2026 using the following keywords in various combinations: “melanoma”, “small lymphocytic lymphoma,” “chronic lymphocytic leukemia,” “collision,” “biphasic,” “biphenotypic,” “concurrent,” “concomitant,” and “coexisting.” All English-language abstracts and full-text articles identified by the search were reviewed. Reports were included if they described histopathologically documented coexistence of malignant melanoma and SLL/CLL within the same tissue specimen or anatomic site, consistent with a collision tumor. Both individual case reports and case series were included. Reports describing melanoma and SLL/CLL occurring in the same patient but at separate anatomic sites, without histopathologic evidence of collision, were excluded. When individual patient-level data were unavailable, cases were included in the literature review and Table 1 using the aggregate information provided in the original publication.

## 3. Case Presentation

A 56-year-old man with a past medical history of psoriasis, CLL, and melanoma presented with a newly developed subcutaneous nodule adjacent to the prior melanoma excision site. On physical examination, the nodule was 27 × 18 mm in size, firm, and the overlying skin was erythematous. No lymphadenopathy or organomegaly was identified. Laboratory studies revealed a white blood cell count of 21.5 × 10^9^/L with an absolute lymphocyte count of 10.7 × 10^9^/L, hemoglobin of 13.5 g/dL, and platelet count of 201 × 10^9^/L; serum beta-2 microglobulin and lactate dehydrogenase levels were within normal limits.

The patient was diagnosed with T1bN0 melanoma of the right flank following wide local excision with sentinel lymph node biopsy about 3 years ago. One year later, the patient was diagnosed with Rai stage 0 CLL based on peripheral blood flow cytometry and complete blood count analysis. Fluorescence in situ hybridization (FISH) studies demonstrated trisomy 12 and del(13q). A mutated immunoglobulin heavy chain variable region (IGHV) rearrangement was identified with IGH somatic mutation analysis. The patient had been managed with active surveillance at 6-month intervals for both conditions. His psoriasis has been well controlled with risankizumab-rzaa, an IL-23 inhibitor.

At this visit, whole-body PET-CT demonstrated intense radiotracer uptake in the right lower flank subcutaneous tissue (SUV max 14.9) and a focal area of uptake in the medial right thigh (SUV max 9), which had no discernible mass on subsequent CT scans. Additional findings included a left lower lobe pulmonary nodule measuring 11 mm with mildly increased uptake (SUV max 4.2). There was no evidence of other organ involvement.

Based on clinical and radiologic findings, metastatic melanoma was suspected. Biopsies of the right flank nodule and medial thigh lesion were performed, both of which confirmed metastatic melanoma. The medial thigh lesion represented a distant soft-tissue metastasis, supporting stage IV disease. Notably, histopathological sections of the thigh lesion also showed an infiltrate of small, monotonous lymphoid cells with clumped chromatin involving adjacent fat and skeletal muscle. Immunohistochemistry showed that the melanoma cells were diffusely positive for SOX10 and MART-1 and showed heterogeneous positivity for cyclin D1 (Figure 1); the lymphoid cells were positive for CD5, CD20, PAX5 and CD23 and negative for CD3, CD10 and cyclin D1 (Figure 2). The Ki-67 proliferation index was approximately 50% in the melanoma and 10% in the SLL/CLL component. Histopathological and immunohistochemical findings were compatible with diagnoses of metastatic melanoma and SLL/CLL (Figure 3), given the known melanoma and CLL history.

Risankizumab-rzaa was discontinued, and the patient was initiated on combination immunotherapy with ipilimumab and nivolumab. The melanoma subsequently tested positive for the *BRAF* V600E mutation, and the patient achieved a partial response after 7 months of treatment, with resolution of the flank lesion and pulmonary nodule.

## 4. Discussion

Published reports of malignant melanoma and SLL/CLL collision tumors remain limited and consist predominantly of individual case reports and small series (Table 1) [7,8,9,10,11,12,13,14,15]. Our literature search identified 16 previously reported cases in which melanoma and SLL/CLL coexisted at the same anatomic site. Among these, Farma et al. [10] identified five patients with metastatic melanoma and SLL/CLL involving the same sentinel lymph node, while Gero et al. [11] reported three patients with both malignancies involving the same lymph-node basin. Because complete individual clinicopathologic characteristics were not available for these patients, the Farma and Gero cases are presented as aggregate cohorts in Table 1.

As summarized in Table 1, 17 cases of melanoma and SLL/CLL collision tumors were identified, including the current case. However, the true frequency of this phenomenon remains unknown and may be underestimated because of underrecognition and publication bias. Individual patient-level clinicopathologic data were available for 9 cases, while Farma et al. and Gero et al. reported 5 and 3 additional cases, respectively, without sufficient individual-level data for detailed analysis. Among the 9 individually characterized cases, 8 patients were male and 1 was female, with ages ranging from 39 to 88 years (median age, 64 years). The temporal relationship between melanoma and SLL/CLL varied: melanoma preceded SLL/CLL in 3 cases, SLL/CLL preceded melanoma in 4 cases, and melanoma and SLL/CLL presented synchronously in 2 cases. The melanoma stage at diagnosis was available for 8 cases and was either stage III (n = 6) or stage IV (n = 2). The anatomic site of collision among the individually characterized cases was most frequently lymph nodes (n = 5), followed by skin (n = 2), combined skin and liver involvement (n = 1), and subcutaneous tissue (n = 1). *BRAF* mutation status was available for 6 cases, 4 of which were mutant. Among the 5 patients with complete treatment and outcome information, only 1 patient was treated with bendamustine and rituximab for SLL/CLL. All 5 patients were treated for melanoma, with chemotherapy (n = 2), a single immune checkpoint inhibitor (ICI; n = 2), or combined ICIs (n = 1) as first-line pharmacological therapy; 2 of them switched to BRAF/MEK inhibitors after a *BRAF* mutation was detected. During the short follow-up, 2 patients died, and 3 patients were alive with disease at the last follow-up.

A collision tumor refers to coexisting neoplasms with different behavioral, genetic, and histopathological features that are sharply demarcated and lack significant tissue admixture [16]. The most frequent combination of collision tumors is MALT (mucosa-associated lymphoid tissue) lymphoma with gastrointestinal tumors, followed by diffuse large B-cell lymphoma with papillary thyroid carcinoma [17,18]. Although cases of synchronously or metachronously diagnosed melanoma and SLL/CLL are not uncommon, collision tumors of these two are exceptionally rare.

Currently, there are three hypotheses regarding the pathogenesis of collision tumors. The simplest, not necessarily the most likely, explanation is the coincidental occurrence of two neoplasms within a common location. SLL/CLL is predominantly a nodal-based lymphoma with a predilection for older men [19], while melanoma commonly affects older, fair-skinned individuals and frequently metastasizes to sentinel lymph nodes; it is not surprising that reported collisions of these two malignancies have occurred predominantly in lymph nodes and in older men. One study reported that the incidence of CLL identified during sentinel lymph node biopsy for melanoma was 4–10 times more frequent than during lymph node evaluation for breast, prostate, and colorectal cancers [10]. The increased incidence of malignant melanoma among patients with SLL/CLL may be partially attributable to more frequent clinical surveillance [20]. Therefore, routine skin surveillance of patients with CLL has been advocated, ideally at the time of SLL/CLL diagnosis, and 6 months following the diagnosis is also acceptable [21]. At least, an annual examination by a dermatologist is strongly recommended [20].

The second hypothesis suggests that a common carcinogenic (genetic or environmental) stimulus may have altered the microenvironment in which two distinct neoplasms arise. Excessive sun exposure and common germline or somatic mutations are proposed causes for the collision of melanoma and SLL/CLL. Though both tumors may harbor *BRAF* mutations, *BRAF* testing has no established therapeutic or prognostic significance in SLL/CLL [22]. However, *BRAF*-mutated SLL/CLL may create a diagnostic pitfall, as the mutation could be incorrectly attributed to the coexisting melanoma. To avoid misclassifying a *BRAF* wild-type melanoma as *BRAF*-mutated, the flank skin lesion, rather than the thigh lesion containing both tumors, was selected for *BRAF* testing in our case. Starza et al. [12] reported that no germline mutation known to predispose to CLL and melanoma was found by conventional cytogenetic and mutational analysis. Moreover, Mansfield et al. [21] suggested that the increased risk, at least for skin cancer, is independent of prognostic factors of CLL, including FISH results, IGHV status, CD38 positivity, etc. This evidence seems to argue against this hypothesis.

The third hypothesis suggests that one tumor may alter the local microenvironment, facilitating the development or homing of another tumor. This hypothesis aligns with the concept that SLL/CLL is an immunosuppressive condition that increases the risk of melanoma, in particular [4]. Moreover, CLL infiltrates have been reported adjacent to other cutaneous neoplasms and at sites of prior inflammation or tissue injury, raising the possibility that local inflammatory or microenvironmental signals may contribute to the recruitment of pre-existing circulating clonal CLL cells [23,24]. In our patient, the diagnosis of melanoma preceded the diagnosis of CLL. However, most CLL patients have an indolent course and may be asymptomatic for a long time until the disease progresses or is incidentally discovered during workups for other diseases. Therefore, we cannot exclude the possibility that the CLL preceded melanoma in our patient. Our patient also had psoriasis and had received risankizumab-rzaa, an IL-23 inhibitor, for at least 3 years. Lam et al. [25] suggested that the coexistence of an autoimmune disease was recognized as a risk factor for increased melanoma-related mortality. Parra-Medina et al. [17] proposed that synergistic interactions between melanoma and lymphoma (involving inflammation and immunosuppression mechanisms) may favor tumor progression and dissemination. However, current evidence does not establish an association between risankizumab and an increased risk of melanoma or SLL/CLL, and its contribution, if any, to the development of the collision tumor in our patient remains unknown [26].

Nevertheless, the sharp histologic interface between the two neoplastic populations establishes a collision pattern but does not demonstrate a causal relationship between the two neoplasms. Of the three proposed mechanisms, we favor the third hypothesis for the collision of malignant melanoma and SLL/CLL, particularly the possibility that a pre-existing CLL clone preferentially homed to the melanoma-associated tumor microenvironment.

In addition to the *BRAF* testing pitfall, another potential diagnostic pitfall in our case is that the collision tumor in the thigh lesion could easily have been missed if a punch biopsy was not done, given the mild elevation in radiotracer uptake. This case highlights the importance of obtaining a tissue diagnosis for suspicious nodules or abnormal PET-CT scan findings, even though a likely cause (metastatic melanoma, in our case) is apparent. This is particularly relevant for lymphadenopathy [27]. Farma et al. [10] pointed out that surgeons and pathologists should recognize that not all grossly enlarged or abnormal lymph nodes in melanoma patients represent melanoma.

Collision tumors pose significant therapeutic challenges due to the lack of formal management guidelines. Management in such cases should prioritize the malignancy with the greater impact on survival, which is typically metastatic melanoma. The advent of immune checkpoint inhibitor (ICI) therapy has significantly improved outcomes in advanced melanoma [20]. Current treatment primarily involves combined immunotherapy with ipilimumab (anti-CTLA-4) and nivolumab (anti-PD-1). BRAF/MEK inhibition with dabrafenib and trametinib is generally reserved for *BRAF*-mutated disease refractory to immunotherapy, partially due to the concern that BRAF inhibitors may promote B-cell malignancies [28]. Notably, the melanoma component of the collision tumor in our case was positive for cyclin D1. Cyclin D1 has been implicated as an oncogenic driver in cutaneous melanoma [29], and its overexpression or *CCND1* amplification has been associated with resistance to BRAF inhibition in *BRAF*-mutated melanoma [30]. Although the diagnostic and prognostic significance of cyclin D1 expression in melanoma remains controversial, higher cyclin D1 expression has been consistently associated with an increased Ki-67 proliferation index [31]. To our knowledge, no prior studies have reported cyclin D1 protein expression or CCND1 amplification status in previously described melanoma and SLL/CLL collision tumors.

## 5. Conclusions

Our case represents a rare cutaneous collision tumor composed of melanoma and SLL/CLL and adds to the limited number of such cases reported in the English literature. The exact pathogenesis of such collision tumors remains unclear, but homing of a pre-existing CLL clone to the melanoma-associated tumor microenvironment might be a plausible explanation. Integrated clinical, radiologic, and histopathologic correlation is essential to avoid diagnostic pitfalls. Clinicians and pathologists should be aware of this unusual association, as recognition of concomitant SLL/CLL may have implications for staging, prognosis, and clinical management.

## Figures and Tables

**Figure 1:**
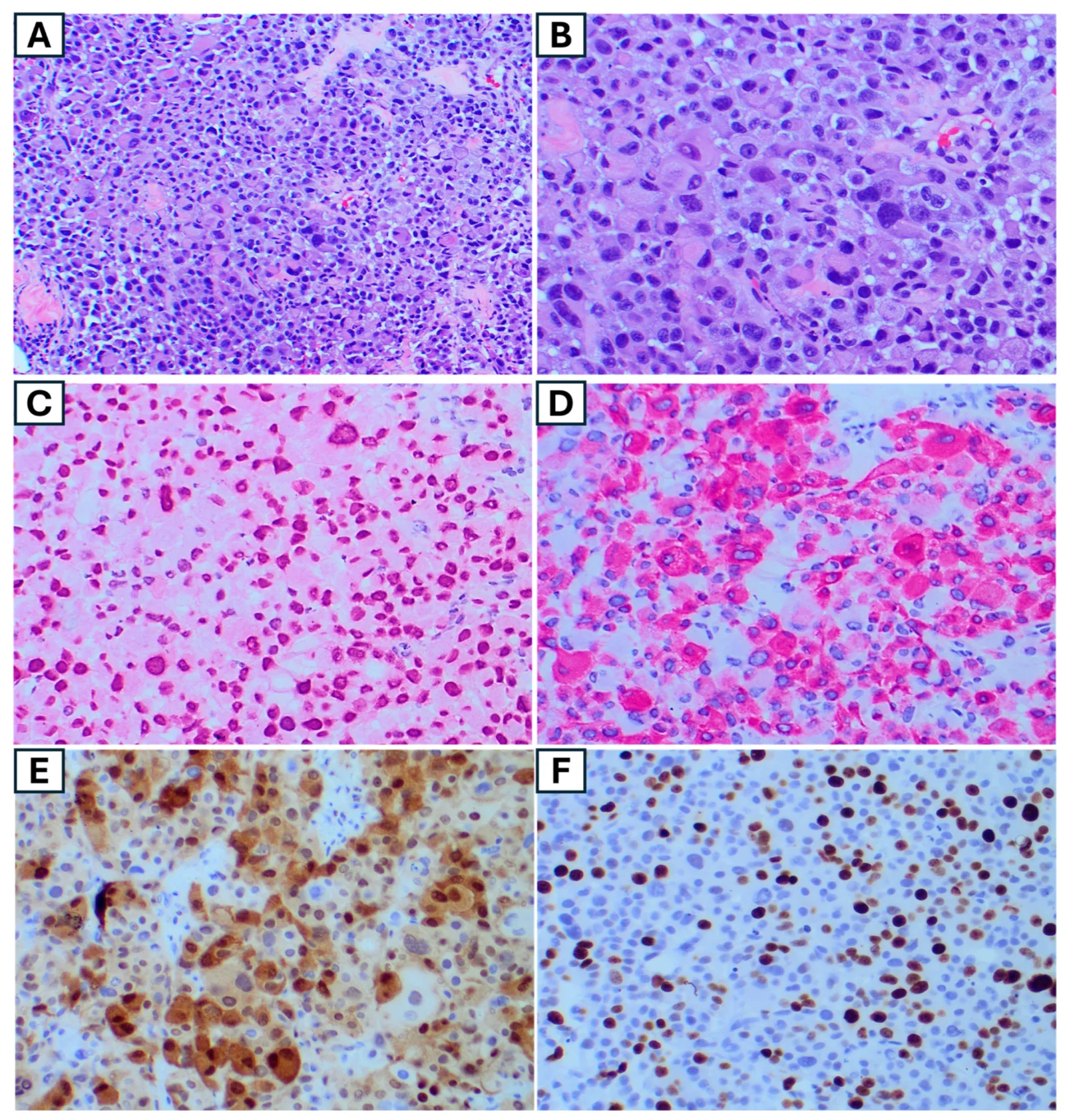
Histopathologic and immunohistochemical features of the melanoma component. (**A**) Low-power H&E showing malignant melanoma involving the subcutaneous tissue (original magnification, ×100). (**B**) High-power H&E showing malignant melanoma composed of atypical epithelioid cells. (**C**) SOX10 immunohistochemistry showing diffuse nuclear staining in the melanoma cells. (**D**) MART-1 immunohistochemistry showing diffuse cytoplasmic staining in the melanoma cells. (**E**) Cyclin D1 immunohistochemistry showing heterogeneous positive staining in the melanoma cells. (**F**) Ki-67 immunohistochemistry showing a proliferative index of approximately 50% in the melanoma cells. Original magnification, ×200 for (**B**–**F**).

**Figure 2:**
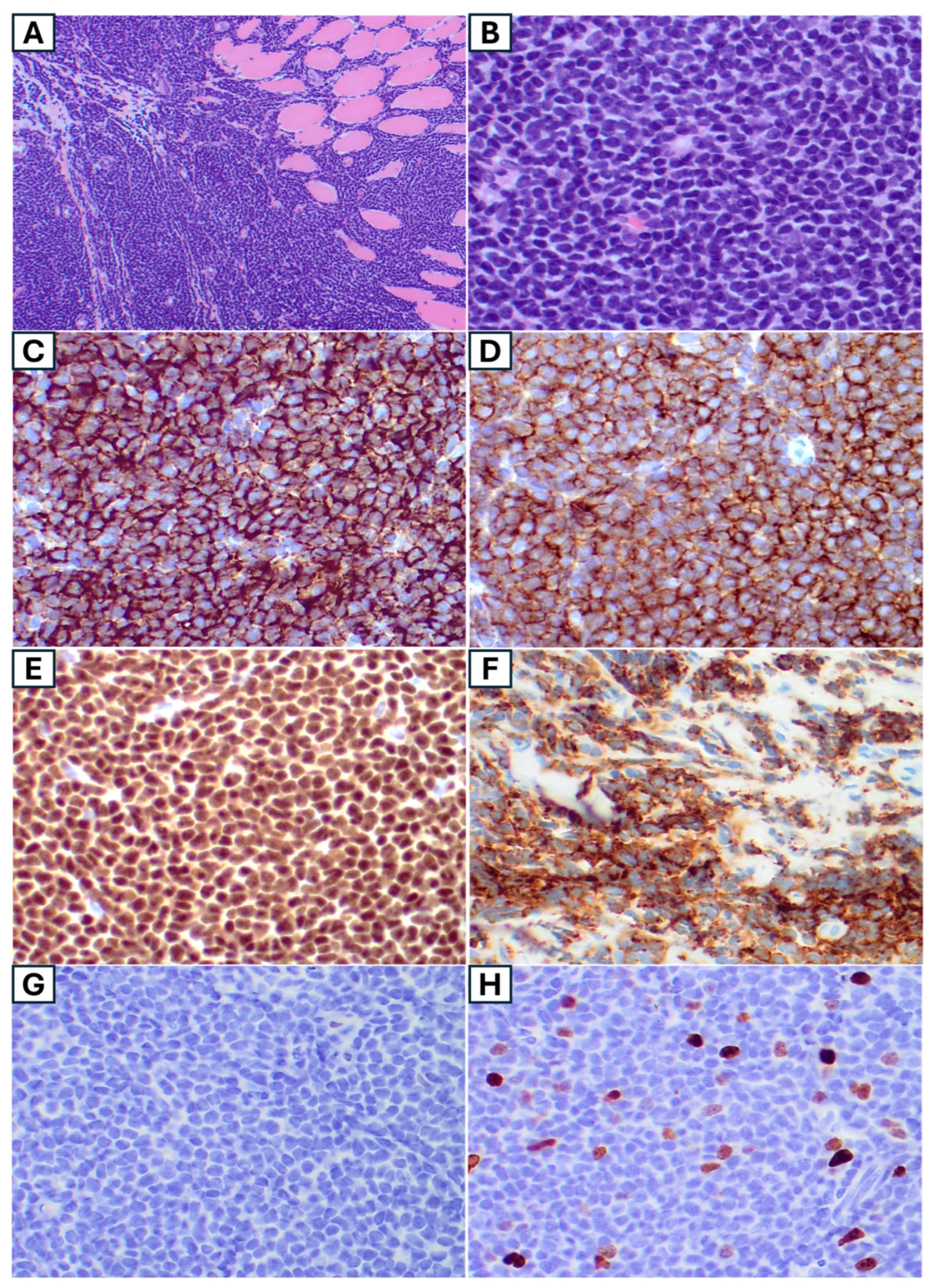
Histopathologic and immunohistochemical features of the SLL/CLL component. (**A**) Low-power H&E showing a dense lymphoid infiltrate involving subcutaneous tissue and skeletal muscle (original magnification, ×100). (**B**) High-power H&E showing small, monotonous lymphoid cells with condensed chromatin. (**C**) CD20 immunohistochemistry showing positive staining in the lymphoma cells. (**D**) CD5 immunohistochemistry showing diffuse membranous staining in the lymphoma cells. (**E**) PAX5 immunohistochemistry showing diffuse nuclear staining in the lymphoma cells. (**F**) CD23 immunohistochemistry showing positive staining in the lymphoma cells. (**G**) Cyclin D1 immunohistochemistry showing negative staining in the lymphoma cells. (**H**) Ki-67 immunohistochemistry showing a low proliferative index of approximately 10% in the lymphoma cells. Original magnification, ×400 for (**B**–**H**).

**Figure 3:**
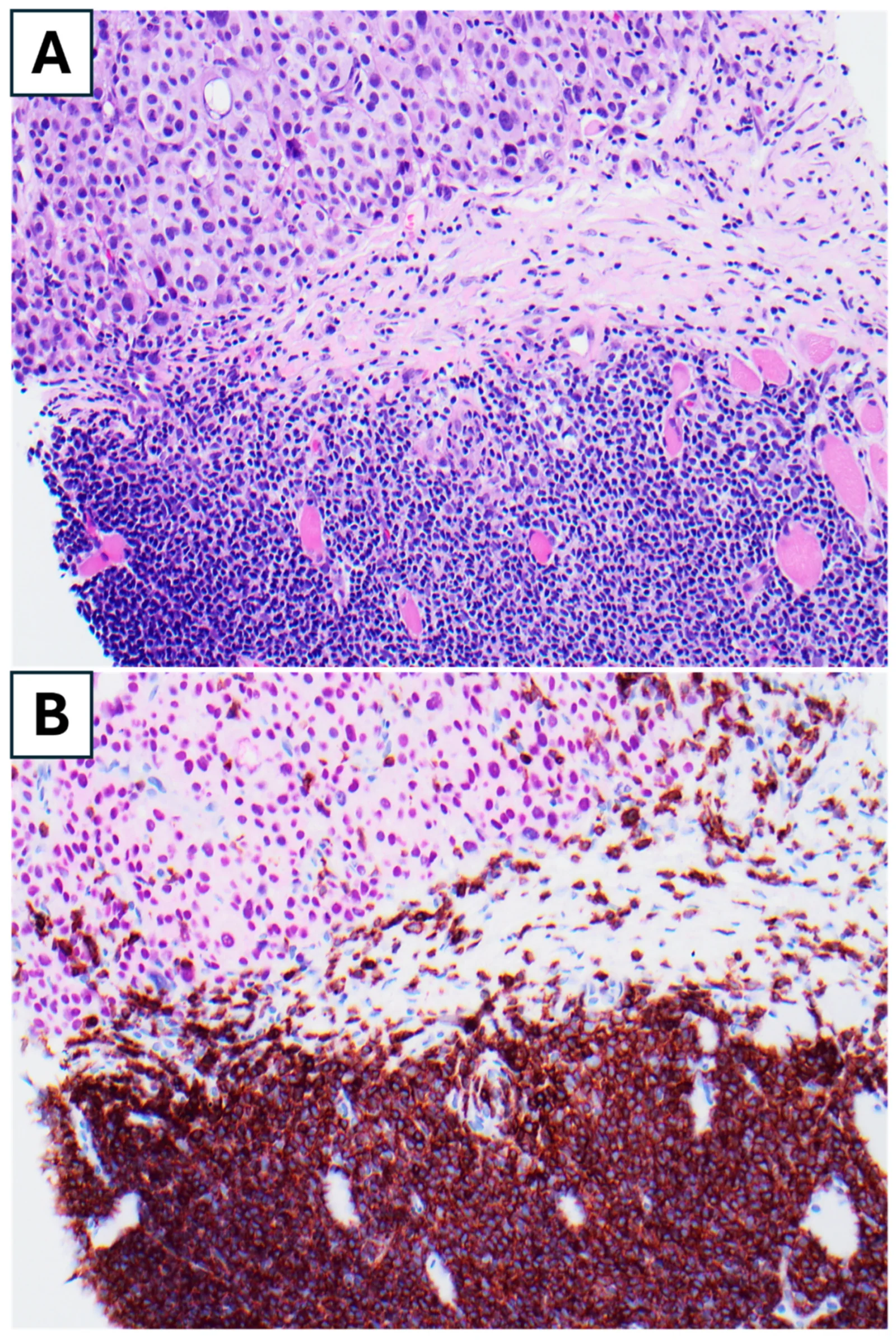
Dual immunohistochemical staining of the melanoma and SLL/CLL collision tumor. (**A**) H&E showing juxtaposed melanoma and SLL/CLL components with a sharp interface. (**B**) Dual SOX10/CD5 immunohistochemistry highlighting the distinct melanoma and SLL/CLL components. Original magnification, ×200.

**Table 1:** Clinicopathologic characteristics, treatment, and outcomes of reported melanoma and SLL/CLL collision tumors.

Author, Year	Cases	Age/Sex	Temporal Relationship	Melanoma Stage	Collision Site	*BRAF*	SLL/CLL Treatment	Melanoma Treatment/Response	Outcome (mo)
El Demellawy et al. [7], 2007	1	58/M	Melanoma first	III	LN	NR	Radiotherapy	NR	NR
Cantor et al. [9], 2010		87/M	SLL/CLL first	III	LN	NR	NR	NR	NR
Dueber et al. [8], 2013	1	39/M	Synchronous	III	LN	Mutant	NR	NR	NR
Farma et al. [10], 2013	5	NR	NR	NR	SLN	NR	NR	NR	NR
Gero et al. [11], 2014	3	NR	NR	NR	LN	NR	NR	NR	NR
Starza et al. [12], 2018	1	58/M	SLL/CLL first	III	Skin	Mutant	Bendamustine, rituximab	Bleomycin: PD → dabrafenib/trametinib: PR	AWD, 9
Diamantopoulos et al. [13], 2020	1	70/M	Melanoma first	III	LN	Mutant	None	Pembrolizumab: PD → dabrafenib/trametinib: PR	DOD, 23
Diamantopoulos et al. [13], 2020	1	76/M	Synchronous	III	LN	Wild type	None	Pembrolizumab: SD	AWD, 6
Dirilenoglu et al. [14], 2020	1	88/M	SLL/CLL first	IV	Skin, liver	Wild type	None	Temozolomide: PD	DOD, shortly
Enslin et al. [15], 2025	1	64/F	SLL/CLL first	NR	Subcutaneous tissue	NR	NR	NR	NR
Current case	1	56/M	Melanoma first	IV	Skin	Mutant	None	Ipilimumab/nivolumab: PR	AWD, 7

SLL/CLL, small lymphocytic lymphoma/chronic lymphocytic leukemia; AWD, alive with disease; DOD, died of disease; PD, progressive disease; PR, partial response; SD, stable disease; LN, lymph node; NR, not reported.

## Data Availability

Data are contained within the article.
